# Comparing organic versus conventional soil management on soil respiration

**DOI:** 10.12688/f1000research.13852.1

**Published:** 2018-03-02

**Authors:** Bence Mátyás, Maritza Elizabeth Chiluisa Andrade, Nora Carmen Yandun Chida, Carina Maribel Taipe Velasco, Denisse Estefania Gavilanes Morales, Gisella Nicole Miño Montero, Lenin Javier Ramirez Cando, Ronnie Xavier Lizano Acevedo

**Affiliations:** 1 Grupo de Investigación Mentoria y Gestión del Cambio, Universidad Politécnica Salesiana, Cuenca, Ecuador; 2 Grupo de Investigación en Ciencias Ambientales, Universidad Politécnica Salesiana, Quito, Ecuador; 3 Ingenería Ambiental, Universidad Politécnica Salesiana, Quito, Ecuador; 4 Administración de Empresas, Universidad Politécnica Salesiana, Guayaqui, Ecuador

**Keywords:** soil respiration, conventional soil management, organic soil management

## Abstract

Soil management has great potential to affect soil respiration. In this study, we investigated the effects of organic versus conventional soil management on soil respiration.  We measured the main soil physical-chemical properties from conventional and organic managed soil in Ecuador. Soil respiration was determined using alkaline absorption according to Witkamp.  Soil properties such as organic matter, nitrogen, and humidity, were comparable between conventional and organic soils in the present study, and in a further analysis there was no statically significant correlation with soil respiration. Therefore, even though organic farmers tend to apply more organic material to their fields, but this did not result in a significantly higher CO2 production in their soils in the present study.

## Introduction

Research related to the benefits of organic management ^1^ has become increasingly important in sustainable agriculture. Organic soil management can contribute to meaningful socio-economic and ecologically sustainable development. Kilcher states that "Organic agriculture reduces the risk of yield failure, stabilizes returns and improves the quality of life of small farmers’ families" ^2^. Soil management has great potential to affect soil respiration, which is an important qualitative indicator of soil microbial activity ^3^. Soil respiration is released as a result of soil organic matter decomposition. The present study aims to investigate the effects of organic versus conventional management on *CO*_2_ production of some Northern Ecuadorian agricultural soils. Our hypothesis was that major soil respiration will be observed in soils under organic management due to the increased amount of applied organic materials.

## Methods

### Sampling sites

Soil samples from 23 organic farms and conventionally managed neighbouring farms were analyzed. In total, 17 sampling sites were located in organic farms, while 6 sampling sites were located in chemical fertilizer-treated areas. The sampling sites were chosen according to proximity of organic and conventionally managed farms in which the same crops are produced. Further details about each of the sampling sites can be found in Table 1. Approximately 1000 g of soil samples of 0–20 cm depth were taken. The following crops were produced in the examined areas: broccoli, potato, tomato and carrot.

**Table 1.** Characteristics of the conventional and organic farms chosen for the present study. Variables are follows: areas of examined lands ( *m*2), Name of crops, soil management (Organic/Conventional), Total crop production (kg), Applied fertilizer (kg), Type of fertilizers, Concentration of NPK, Concentration of NPK, Amount of NPK (Kg), GPS coordinates of the examined lands.

Farmer’s code		Crop	Solid fertilizers	Area of land m2	Total crop production (Kg)	Fertilizer application rate on total crop production (Kg)	Concentration of NPK (%) in each fertilizer solid			Amount of NPK in kg			liquid fertilizer	Fertilizer application rate on total crop production (Kg)	Concentration of NPK (%) in each liquid fertilizer			Amount of NPK in Kg			GPS coordinates	
							N	K	P	N	K	P			N	K	P	N	K	P	latitude	length
OB1	Broccoli	Agroecological	Compost	60.38	315	95.25	0.53	0.6345	1.322	0.504825	0.60436125	1.259205	Biol	2.63975	0.2428	0.8183	0.3061	0.00640931	0.02160107	0.008080275	O804800	OOO3519
OB2		Agroecological	Bocashi	118.2	576	268.03	0.17	0.4013	0.071	0.455651	1.07560439	0.1903013	Biol	185	0.2	1.0816	0.0148	0.37	2.00096	0.02738	O809419	OOO6402
OB3		Agroecological	Compost	9	79.2	20.2	0.3	0.3891	0.1221	0.0606	0.0785982	0.0246642	biol	30	0.14	0.0075	0.467	0.042	0.00225	0.1401	O809136	OOO3476
OB4		Agroecological	Bocashi	144	600	300	0.5	0.8667	0.1271	1.5	2.6001	0.3813	Biol	211.18	0.24	0.4033	0.0958	0.506832	0.85168894	0.20231044	O804806	OOO3527
OB5		Agroecological	Bocashi	56	326.7	101	0.43	0.4427	0.5081	0.4343	0.447127	0.513181	Biol	150	0.2202	0.2862	0.0735	0.3303	0.4293	0.11025	O811423	OOO3176
CB1		Conventional	18460	2511.6	8316	82.21654	18	0	46	14.7989772	0	37.8196084									O805608	OOO1169
			OO60			33.33904	0	60	0	0	20.003424	0										
			Triple 15			44.4521	15	15	15	6.667815	6.667815	6.667815										
			UREA			2.1	46	0	0	0.966	0	0										
OT1	Tomato	Agroecological	Gallinaza	322.766	0.0322766	1550	0.59	0.6815	0.8673	9.145	10.56325	13.44315	Biol	78.51387307	1.09	1.5659	0.5374	0.85580122	1.22944874	0.421933554	0811193	0006955
			Compost			1550	0.89	2.5875	0.6949	13.795	40.106	1.077										
OT2		Agroecological	Humus 1	202.4	0.02024	2470	1.24	2.9429	1.0828	30.628	72.68963	26.74516	Biol	2086.4584	0.26	0.3443	0.2216	5.42479184	7.18367627	4.623591814	0809214	0003617
			Humus 2			2470	0.66	0.7458	0.5232	16.302	18.42126	12.92304										
			Bocashi			2470	0.8	1.2478	0.6486	19.76	30.821	16.02										
			Bocashi negro			2470	1.29	1.0581	0.2705	31.863	26.135	6.681										
OT3		Agroecological	Compost	250.912	0.0250912	6.38	0.39	0.8731	0.2064	0.024882	0.05570378	0.001316832	Biol	11964.3692	0.220045	0.073448586	0.2859998	26.3269962	8.78766	34.218072	0811429	0003184
			Bocashi			6.38	0.43	0.5081	0.4427	0.027434	0.033	0.028										
CT1		Conventional	Nitrogen Magnesium	847.132	0.0847132	41.32	10.7	0	0	3.79	0	0									0809021	0002732
			Ultrasol K			41.32	13	46	0	5.3716	19.0072	0										
CT2		Conventional	8-20-20	4827.69	0.482769	99.43	8	20	20	7.95	19.89	19.89									0805316	0001139
			MAP			99.43	12	0	61	11.93	0	60.65										
			EC FERTILIZER			41.43	15.5	0	0	5.14	0	0										
CT3		Conventional	Florone	1234.865	0.1234865	5.668635843	1	9.5	5	0.056686358	0.538520405	0.283431792									0805312	0001138
			Nitrofoska foliar			40.49025602	8	24	12	3.24	9.71766145	4.8588307										
OP1	POTATO	Agroecological	Compost	116	408.16	1360	0.53	0.6345	1.322	7.208	8.629	17.979	Biol	1.0559	0.2428	0.8183	0.3061	0.00256373	0.00864043	0.00323211	804851	3376
OP2		Agroecological	Gallinaza	88.2	136	50	3.14	4.3752	6.0922	1.57	2.1876	3.0461	Biol	84.472	0.17	0.4013	0.071	0.1436024	0.33898614	0.0599712	809414	6481
			Cal Agrícola			9.0719	x	x	x	x	x	x										
OP3		Agroecological	Bocashi	69.9	181.4	1500	0.38	0.7695	0.4772	5.7	11.5425	7.158	Biol 1	12.6708	0.22	0.3619	0.013	0.02787576	0.04585563	0.001647204	808161	3438
													Biol 2 microorga.	6.3354	0.13	0.2065	0.0065	0.00823602	0.0130826	0.000411801		
OP4		Agroecological	Compost	13.17	45.35	4.5359	0.91	0.4283	0.865	0.04127669	0.01942726	0.03923554	Biol	1.58385	0.18	0.224	0.0387	0.00285093	0.00354782	0.00061295	808225	3496
CP1		Conventional	Harvest waste	10 000	13610	3000	x			x	x	x									810311	5670
			103010			750	10	10	30	75	75	225										
			18460			250	18	46	0	45	115	0										
			Stimufolk			4	11	38	5	2.2	7.6	1										
			Agricare			4	19	19	19	9.5	9.5	9.5										
OC1	Carrot	Agroecological	Compost	92.97	2045	146.66	0.53	0.6345	1.322	0.777298	0.9305577	1.9388452	Biol	4.06	0.2428	0.8183	0.3061	0.00985768	0.03322298	0.01242766	17N 0804805	0003544
OC2		Agroecological		15.645	156		0	0	0	0	0	0	Biol	2	0.23	0.007	0.0181	0.0046	0.00014	0.000362	17 N0811449	0003795
OC3		Agroecological	Bocashi	9	72	1.35	0.38	0.7695	0.4772	0.00513	0.01038825	0.0064422	Biol (1)	1.64	0.22	0.3619	0.013	0.003608	0.00593516	0.0002132	17 N 0808284	0003066
													Biol (2)	0.82	0.13	0.2065	0.0065	0.001066	0.0016933	0.0000533		
OC4		Agroecological	Bocashi	11.2	246	23.3	0.5	0.8667	0.1271	0.1165	0.2019411	0.0296143	Biol	16.425	0.24	0.4033	0.0958	0.03942	0.06624203	0.01573515	17 N 0804808	0003504
OC5		Agroecological	Compost	60	1500	134.6	0.3	0.3891	0.1221	0.4038	0.5237286	0.1643466	biol	200	0.14	0.0075	0.467	0.28	0.015	0.934	17 N O809136	0003548
CC1		Conventional		176.56	108								Biofertilizante (lombriz)	60	0.32	0.3963	0.4595	0.192	0.00126816	0.001820999	17 N 0805383	0001613
			Nitrato de Calcio			0.13	15	0	0	0.0195	0	0										
			Fosfato Monoamonico			0.07	11	0	52.5	0.00733333	0	0.035										
			Nitrato de Potasio			0.07	13		44	0.00866667	0	0.02933333										

### Soil properties

Soil moisture content was determined gravimetrically, drying the soil at 105°C for 24 hours according to Fernández *et al.* (2008) ^4^. Soil texture was measured using sodium hexametaphosphate (( *NaPO*_3_) _6_) according to Bouyoucos (1962) ^5^. To measure the soil chemical properties, the samples were sieved through a 2mm mesh and pre-incubated at 25° for 72 hours. Soil pH in distilled water (soil/water, 1/2.5, w/w) was determined according to Karkanis (1991) ^6^. In addition, we measured the electrical conductivity (EC) using a glass electrode according to Karkanis (1991) ^6^. Cylinder volume was determined according to Agostini *et al*. (2014) ^7^. Soil organic matter was determined according to Walkley and Black (1934) ^8^. We measured the phosphorous content according to Olsen (1954) ^9^. The Sand/Silt/Clay ratio was determined by Bouyoucos’s method (1936) ^10^, while the cation exchange capacity was determined according to ISO 11260 (1994) ^11^ protocol.

### Soil respiration

The experiment was applied at 25° *C*. 0, 1 *M* NaOH (10 *ml*) was placed in laboratory bottles (250 *ml*), a sterile gauze pad were filled with 10 g of soil sample according to Witkamp (1966) ^12^. After 10 days, the amount of *CO*_2_ was subsequently measured by standardized titration against 0.1 *N* HCl using firstly phenolphthalein and then methyl orange indicator according to Witkamp (1966) ^12^.

The below formula was applied to calculate soil respiration:

*m*( *CO*_2_) = *VxNx*22 *CO*_2_

And *CO*_2_ production (for 10 days):

*mg*( *CO*_2_) * 100 *g* – 1 * 10 *day* – 1 = methyl orange factor * HCI – *phenolphthaleinloss*) * *NAOH* *factor* * 2, 2 * *Moisture multiplication factor*

where

$Moisture\;multiplication\;factor=\frac{\left(moisturecontent\%+100\right)}{100}$

We determined the volume of the examined soils (counting with 0 – 20 cm depth) using topsoil calculator tool ( https://www.tillersturf.co.uk/topsoil-calculator). The results of soil respiration was then estimated in kg( *CO*_2_)/ha/day.

### Statistical analysis

To evaluate the behavior within results, two types of test were performed: i) Student’s t-test for comparing means between conventional and organic crop systems in terms of soil respiration (kg/CO2/ha/day), organic matter (%) and nitrogen (%). Furthermore, Person’s and Spearman’s correlation were fixed in order to test data covariation and correlation. ii) ANOVA was used to compare conventional and organic crop system and the type of crop harvested in the sampling site.

## Results

The results of soil respiration from areas of organic and conventional soil management are comparable ( Dataset 1).

**Figure 1.**
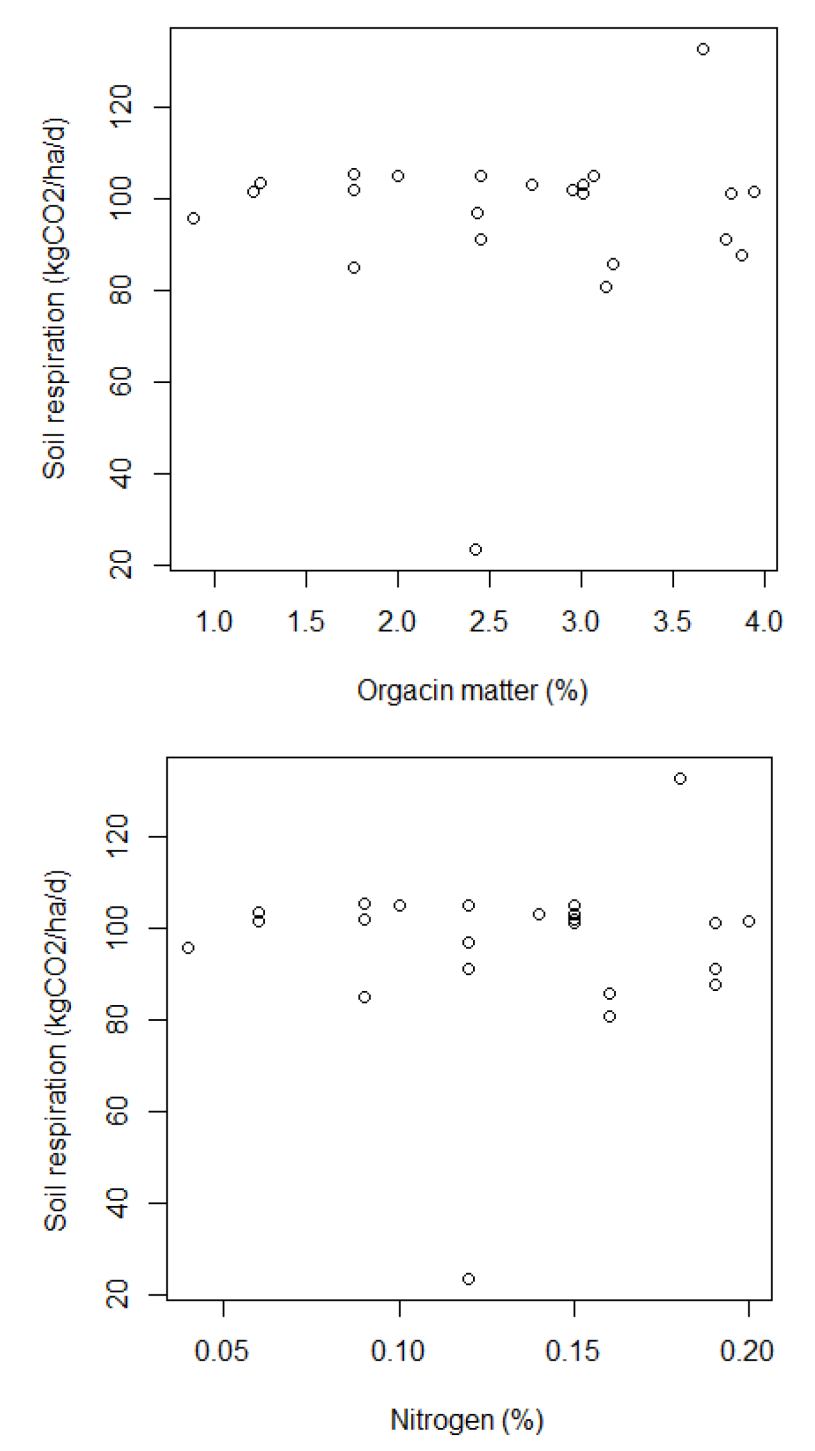
Soil respiration compared with organic matter and nitrogen in soil.

For soil respiration, conventional soil mean was 88.50 and organic mean was 98.64, showing and increment around 10%. However, there were no statistically significant differences between group means as determined by one-way ANOVA (p =0.15), comparing conventional and organic systems. Pearson‘s and Kendell‘s tests have showed no correlation. Soil respiration correlation coefficient with organic matter was lower than 0.05 and with nitrogen content was lower than 0.12. This analysis did not consider the differences between conventional and organic systems ( Figure 1).

**Figure 2.**
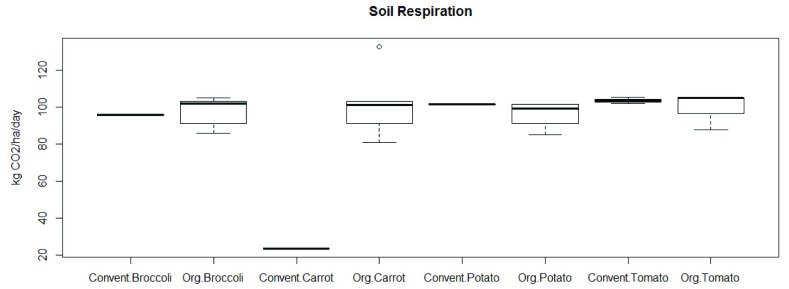
Boxplots showing alterations within crop systems and crop harvested in the zone.

There were statistically significant differences between group means as determined by one-way ANOVA (p < 0.05), comparing crop types. Furthermore, a post hoc test (Duncan) was fixed. There was only one crop (carrot) in conventional system (odds lower than 0.05) that differs drastically from the others, as pointed out in ( Figure 2).

Considering soil characteristics (pH, CIC, K, and Electric conductivity), Student’s t-test was applied to identify differences between conventional and organic systems. Only the characteristics from carrot crop systems (conventional or organic) have shown differences in terms of means (p < 0.05). Furthermore, the mean of conventional crop system was lower in every characteristic evaluated. Besides, these results were in congruence with Figure 2, leading us to believe that the cropping system has no influence on soil respiration, which is in contrast to the influence that soil characteristics have over soil respiration in this study.

Raw data for various parameters calculated in conventional and organic managed soils Parameters as follows: pH, Organic material (percentage), Total Nitrogen (percentage), Match (mg/kg), Potassium (cmol/kg), Electrical conductivity (dS/m), CIC (cmol/kg), Soil moisture content (percentage), Sand (percentage), Silt-limo (percentage), Clay (percentage), Texture (class), Soil respiration (kg/CO2/ha/day). Click here for additional data file. Copyright: © 2018 Mátyás B et al. 2018 Data associated with the article are available under the terms of the Creative Commons Zero "No rights reserved" data waiver (CC0 1.0 Public domain dedication).

## Conclusions

Organic farmers tend to apply more organic material to their fields, but this did not result in a significantly higher *CO*_2_ production in their soils. The difference between organic and conventional soils (10% in mean) is not enough to conclude that the soil respiration under these two systems was different, considering the analysis of their variance.

Soil properties like organic matter, nitrogen, and humidity, were comparable between conventional and organic soils in the present study, and in a further analysis there was no statically significant correlation with soil respiration. However, biological significance should be investigated in a posteriori research including microbial community profile of the soil and specific interactions in highlands (over 2500 m.a.s.l.).

## Ethics

Oral consent was obtained from the farmers for the collection of soil samples from their land. Their only request was to inform them about the results of the soil characteristics, that we have already done personally on 9 November, 2017.

## Data availability

The data referenced by this article are under copyright with the following copyright statement: Copyright: © 2018 Mátyás B et al.

Data associated with the article are available under the terms of the Creative Commons Zero "No rights reserved" data waiver (CC0 1.0 Public domain dedication). 

**Dataset 1: Raw data for various parameters calculated in conventional and organic managed soils.** Parameters as follows: pH, Organic material (percentage), Total Nitrogen (percentage), Match (mg/kg), Potassium (cmol/kg), Electrical conductivity (dS/m), CIC (cmol/kg), Soil moisture content (percentage), Sand (percentage), Silt-limo (percentage), Clay (percentage), Texture (class), Soil respiration (kg/CO2/ha/day). DOI, 10.5256/f1000research.13852.d195529 ^13^
