# Supplementary material for: Comparing organic versus conventional soil management on soil respiration
Source: F1000Res. 2018 Mar 2;7:258. [Version 1] doi: 10.12688/f1000research.13852.1 (PMC5861514; doi:10.12688/f1000research.13852.1)
Supplement: Peer review history

# Peer review: Referee response for version 1

**DOI:** 10.5256/f1000research.15056.r31654

**Published:** 2018-03-19

**Authors:** Ankit Singla

**Affiliations:** 1 Department of Industrial Microbiology, Sam Higginbottom Institute of Agriculture, Technology and Sciences, Allahabad, Uttar Pradesh, India

The submitted manuscript by Bence et al. is good work which is suitable for publication in F1000 research. Authors have compared the organic practices and conventional practices, and compared their effects on soil respiration which is very important aspect. Standard methodologies were followed which ensures reproducibility of the results. The findings were subjected to the statistical analysis and conclusion drawn nicely.

 However, I have below suggestions for improvement which may be considered as minor revisions:

- "Physical-chemical" could be replaced by "Physico-chemical" throughout the manuscript.
- In abstract, word "statically" should be replaced by "statistically"
- In result, "showing and increment around 10%." should be "showing an increment around 10%."
- The discussion could be added more so that the findings of the study will become stronger.

I have read this submission. I believe that I have an appropriate level of expertise to confirm that it is of an acceptable scientific standard.

# Peer review: Referee response for version 1

**DOI:** 10.5256/f1000research.15056.r31652

**Published:** 2018-03-15

**Authors:** Anita Jakab

**Affiliations:** 1 National Agricultural Research and Innovation Centre, National Agricultural Research and Innovation Centre, National Agricultural Research and Innovation Centre, Újfehértó, Hungary

This article worked at the differences between organic and conventional soil management. This research examined an important and topical issue especially the soil respiration under changing plant and soil conditions.

Introduction and methods

 The research investigated 23 soil samples in Ecuador. The samples were located from organic (17 samples) and conventionally managed neighboring farms (6 samples). In the research trials broccoli, potato, tomato and carrot were applied as test plant. Soil properties were measured after 1000 g soil samples of 0-20 cm depths of soil were taken in every picked area. The soil moisture, texture, pH, electrical conductivity, cylinder volume, organic matter, phosphorus content, sand/silt/clay ratio and cation exchange capacity, and the soil respiration were analyzed in laboratory.

 The values of the soil parameters are presented in a dataset, which inform about the important soil parameters especially the calculated soil respiration in kg (CO _2_)/ha/day). The protocols (description of the tests) are clear and traceable, especially the formula to calculate soil respiration.

 The study describes the applied type of fertilizers especially the concentration of NPK fertilizers.

Comment on the Methods

 - The sampling time and vegetation status are important for the evaluation, this information is missing in the study. If it’s possible, describe the followings: When the soil sampling happened? What was the state of the vegetation of test plants?

 - A bit more detail of the soil properties inform us about the actual soil status. The studied soils are classified as sandy textured soil, according to the soil classification (Franco Arenoso). The most typical parameters of the samples are the following: high sandy texture, neutral pH, good/very good organic matter-nitrogen and phosphorus content, 10-20% moisture content. I suggest describing it in the Methods.

Results

 The results of the study are described with sufficient statistical analysis. It also describes the statistically significant/not significant results. There were solely statistically significant differences between crop types (for soil respiration by one-way ANOVA correlation test).

 - The Figure 1 contains a typographical error (Orgacin matter instead of Organic matter).

 - It may be more informative, if you use a line diagram instead of dot diagrams in the first figure.

 - The Figure 2 include the soil respiration values in kg CO _2_/ha /day, which would be more clear with the average values.

Conclusion

 The results have briefly evaluated and conclusions straightforward formulated. I quite agree with observations of the study that emphasizes the importance of further microbiological studies.

I have read this submission. I believe that I have an appropriate level of expertise to confirm that it is of an acceptable scientific standard.
